# Effect of Vitamin D, Calcium and Multiple Micronutrients Supplementation on Lipid Profile in Pre-menopausal Bangladeshi Garment Factory Workers with Hypovitaminosis D

**Published:** 2014-12

**Authors:** Md. Zahirul Islam, Abu Ahmed Shamim, Abu Ahmed, Mohammad Akhtaruzzaman, Merja Kärkkäinen, Christel Lamberg-Allardt

**Affiliations:** ^1^Calcium Research Unit, Department of Food and Environmental Sciences (Nutrition), PO Box 66, 00014, University of Helsinki, Finland; ^2^Institute of Nutrition and Food Science, University of Dhaka, Dhaka 1000, Bangladesh; ^3^Food Security Nutritional Surveillance Project (FSNSP), BRAC Institute of Global Health, BRAC University, icddr,b Main Building, Level 6, Mohakhali, Dhaka 1212, Bangladesh

**Keywords:** Female garment workers, HDL-cholesterol, LDL-cholesterol, Total cholesterol, Triacylglycerol, Vitamin D intervention, Bangladesh

## Abstract

Elevated total cholesterol and low-density lipoprotein cholesterol in sera are both well-known risk factors of coronary heart disease. Adequate vitamin D status is important for optimal function of many organs and tissues of our body. There is continuing controversy about the effect of adequate vitamin D consumption on serum lipids and lipoproteins. The present study assessed the effect of vitamin D, calcium and multiple micronutrients supplementation on the lipid profile in Bangladeshi young female garment factory workers who have hypovitaminosis D. This placebo-controlled intervention trial conducted over a period of one year randomly assigned a total of 200 apparently healthy subjects aged 16-36 years to 4 groups. The subjects received daily supplements of 400 IU of vitamin D (VD group) or 400 IU of vitamin D+600 mg of calcium lactate (VD-Ca group), or multiple micronutrients with 400 IU of vitamin-D+600 mg of calcium lactate (MMN-VD-Ca group), or the group consuming placebo (PL group). Serum concentrations of lipid and lipoprotein, 25-hydroxyvitamin D (25OHD) and intact parathyroid hormone (iPTH) were measured at baseline and after one year of follow-up. No significant changes in the serum levels of total cholesterol (TC), low-density lipoprotein cholesterol (LDL-C), high-density lipoprotein cholesterol (HDL-C), LDL-C/HDL-C ratio were observed in the supplemented groups compared to the placebo group. Supplementation had a positive effect (p<0.05) on very low-density lipoprotein cholesterol (VLDL-C) and triacylglycerol (TAG). A negative correlation between changes in serum iPTH and HDL-C was observed, which indicated that subjects with the greatest decline in S-iPTH had the greatest increase in HDL-C. The results suggest that consumption of adequate vitamin D with calcium or MMN for one-year may have no impact on serum lipid profile in the subjects studied. Longer-term clinical trials with different doses of supplemental vitamin D are warranted in evaluating the effect of intervention.

## INTRODUCTION

Vitamin D deficiency causes metabolic bone disease and increases the risk of many common chronic diseases (1). Adequate vitamin D status is important for optimal function of many organs and tissues of our body, including cardiovascular system (2). Vitamin D deficiency has been found to be associated with dyslipidaemia, cardiovascular diseases (CVDs), and mortality (3-6).

Elevated total cholesterol (TC) and low-density lipoprotein cholesterol (LDL-C) in the sera are both well-known risk factors of coronary heart disease (CHD) whereas elevated concentrations of high-density lipoprotein cholesterol (HDL-C) appear to protect against CHD. Increased concentrations of serum triglycerides are also associated with an increased risk of CHD (7). Obesity and type 2 diabetes occur at epidemic rates globally and atherosclerotic CVD remains one of the leading causes of mortality and morbidity (8). Recent epidemiological data suggested that subjects with serum 25OHD levels of ≥75 nmol/L had a 6.1-fold reduced risk of incident hypertension compared to vitamin D deficient (serum 25OHD level <35 nmol/L) subjects (9). One probable explanation could be the effect of vitamin D on serum lipids, one of the major risk factors of CVD, or vitamin D is a vaso-active agent and may play a protective role in the development of atherosclerosis.

However, based on the intervention trials, the available data do not support unanimously the effect of adequate vitamin D supplementation on serum lipids. Only limited data suggested that CVD risks could be reduced with vitamin D supplements at moderate to high doses and the investigators stressed further research in this field (10,11). In fact, data are scarce about the impact of adequate vitamin D consumption—whether supplementation with vitamin D would improve cardiovascular risk factors is not studied extensively. This study assessed the effect of vitamin D, calcium and multiple micronutrients supplementation on serum lipids and lipoproteins in a representative sample of Bangladeshi low-income pre-menopausal female garment workers with hypovitaminosis D.

## MATERIALS AND METHODS

### Study setting and subjects

The study site was an export-oriented garment factory located at Mirpur in Dhaka city, belonging to Standard Group Bangladesh. The participants were mainly young women from low-income rural families, who had migrated to the city for employment at least two years earlier. Subjects lived in an underdeveloped area in low-cost accommodation. They worked dawn to dusk, 7 days a week. The eligibility criteria for inclusion of subjects comprised: no history of serious diseases, no history of medication known to affect bone metabolism, no current pregnancies, no lactation within the previous three years, and residing in the city for at least two years. The fieldworkers explained the objectives of the study to the subjects in an understandable way. Written informed consent was obtained from both interested subjects and the authority of the garment factory before initiating the study. The study protocol was approved by the Ethical Committee of the Faculty of Agriculture and Forestry, University of Helsinki. During the field study in Bangladesh, we also followed the ethical guidelines of the University of Dhaka.

### Study design

**Figure 1.**
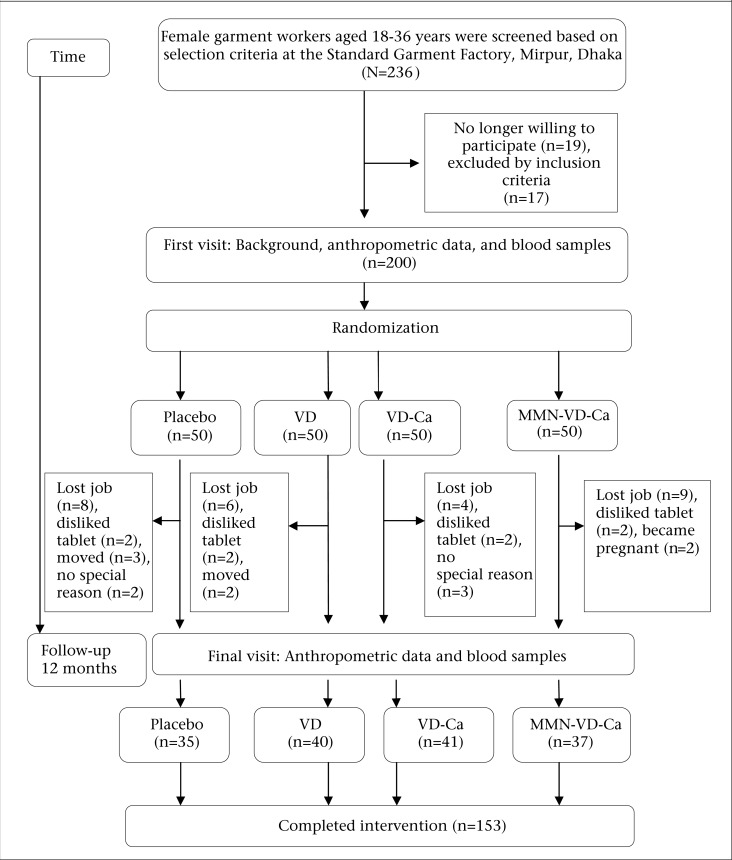
Flowchart of the number of subjects recruited and dropping out: VD, VD-Ca, and MMN-VD-Ca

The study comprised a one-year, placebo-controlled, randomized intervention trial. The assigned 200 subjects met all of the inclusion criteria. Participants were randomly assigned to one of the four groups with blocks of equal size after their eligibility criteria had been fulfilled. Randomization was carried out by a person who was not involved in the project. The subjects were provided with daily supplements of 400 IU of vitamin D (VD group), 400 IU of vitamin D+600 mg of calcium as calcium-lactate (VD-Ca group), multiple micronutrients with 400 IU of vitamin D+600 mg of calcium as calcium-lactate (MMN-VD-Ca group), or a placebo (placebo group). The recruitment and randomization processes are presented in Figure 1. A total of 50 subjects in each group were selected for supplementation. The number of subjects who completed intervention in different groups were: VD (n=40), VD-Ca (n=41), MMN-VD-Ca (n=37) and Placebo (n=35). The total dropout percentage was 23.5. The compositions of supplements comprising oral calcium tablets (‘G-Calcium’ from Gonoshasthaya Pharmaceuticals Ltd), MMN tablets (‘Aristovit M’ from Beximco Pharmaceuticals Ltd., Bangladesh, containing 15 micronutrients) and vitamin D tablets (‘Minisun’ from Oy Verman AB, Jarvenpaa, Finland) are presented elsewhere (12). Both calcium and vitamin D placebos were donated by the same companies and were identical to the active tablets.

Subjects received their supplements daily from the field assistants for 12 months. The field assistants arrived at the garment factory every day (7 days a week) before the lunch break. Tablets were given to the subjects during their lunch break, inside the garment factory, under close observation of the field assistants. The field assistants always ensured that the tablets were swallowed with a glass of water and maintained a written daily record of supplement consumption by each subject. In case a subject was absent from work, supplements were given to their colleagues to deliver to their co-workers for consumption in the evening at home.

### Laboratory measurements

Blood samples were collected between 8:30 and 10:00 am after an overnight fasting. Five mL of venous blood were drawn using disposable syringes. The serum was separated and preserved at −20 °C. Finally, the serum samples were transported to Helsinki in a special container with dry ice and preserved in the freezing room of the Division of Nutrition, Department of Food and Environmental Sciences, University of Helsinki, at −20 °C until analysis.

Serum total cholesterol (TC), high-density lipoprotein cholesterol (HDL-C), and triacylglycerol (TAG) were measured with an automated KoneLab spectrophotometer (Thermo Clinical Labsystems Ltd., Espoo, Finland), using routine methods. The concentration of serum VLDL-C was calculated by using the equation VLDL-C=TAG×0.45 and the concentration of serum LDL-C by using the equation LDL-C=TC–HDL-C–VLDL-C. Serum 25-hydroxyvitamin D was used in evaluating vitamin D status. The concentration was determined by the enzyme-immunoassay method with kits from OCTEIA (IDS, Boldon, UK). The laboratory is a partner of the Vitamin D External Quality Assessment Scheme (DEQAS, www.DEQAS.org.uk). The intra-and inter-assay CVs were 5.4% and 7.0% respectively. Serum intact parathyroid hormone (S-iPTH) level was measured with a commercial two-site immunoenzymometric assay (OCTEIA, IDS, Boldon, UK), with 10-65 ng/L as a reference range. Intra- and inter-assay CVs for S-iPTH were 3.5% and 5.6% respectively. The laboratory analysis was carried out at the Department of Food and Environmental Sciences, University of Helsinki. The vitamin D deficiency, insufficiency, and sufficiency were defined as S-25OHD levels of <50 nmol/L, 50-75 nmol/L, and >75 nmol/L respectively. The secondary hyperparathyroidism was defined as S-iPTH levels of >65 ng/L. All measurements were taken at baseline and after 12 months of follow-up. The study was conducted from April 2004 to April 2005.

### Other data

Anthropometric, socioeconomic and background data were collected at baseline and after one year of follow-up. A questionnaire was used for obtaining information on monthly income, level of education, age at menarche, duration of residence in the city, daily time spent outdoors, etc. Standing height was measured with a wall-mounted scale to the nearest 0.5 cm. Body-weight was measured without shoes and with light clothing on a portable weighing scale to the nearest 0.5 kg. We used the classifications of body mass index (BMI) [weight (kg)/height (m)^2^] recommended by the World Health Organization (13).

### Statistical analysis

Statistical analyses were carried out with SPSS (version 15.0) for Windows (SPSS, Chicago, IL, USA). The normal distribution of variables was assessed with a Kolmogorov-Smirnov test. If normality was not present, logarithmic transformations were made before further analysis. Serum lipid data and other selected variables were tested with both ANOVA and ANCOVA to show the effect of confounding factors. Covariates were also noted in each analysis. The post-hoc analyses were performed with Tukey's Honestly Significant Different (HSD) test and Dunnett's test. A multiple linear regression model with age, BMI, serum calcium, serum iPTH, and creatinine as covariates was used in evaluating S-25OHD as an individual predictor of serum lipids and lipoproteins. Correlations were evaluated with Pearson's correlations coefficient—*r*. All data are reported as means±standard deviations; p<0.05 was considered significant.

## RESULTS

A total of 153 subjects completed the one-year intervention trial out of 200 subjects selected at the baseline, and 23.5% of the participants (47 subjects) dropped out for some reasons not related to the study. The number of subjects at various stages is shown in Figure 1. We observed no significant changes in socioeconomic status, level of education, physical activity, lifestyle, daily time spent outdoors in the sunshine, and most anthropometric characteristics between the supplemented groups at baseline and after supplementation (all data not shown). No significant group difference was present with respect to BMI both at baseline and post-supplementation. A summary of the baseline descriptive, biochemical characteristics of the study population completing the trial is shown in Table 1.

There was no significant difference in serum lipid status among placebo and supplemented groups at baseline. More than 92% of the subjects had serum T-C <5.0 mmol/L at baseline, which is generally considered the borderline higher limit of desired range. Serum T-C levels of >5.0 mmol/L were observed in 12 subjects in four groups—three in the placebo group, six in the VD group, two in the VD-Ca group, and one in the MMN-VD-Ca group. We observed that 99% of the subjects in randomly-assigned groups had very low HDI-C level (<1.0 mmol/L) and the prevalence of low HDI-C level was not changed significantly even after one year of supplementation.

No significant (p>0.05) group differences were observed between the four groups both at baseline and after 12 months of follow-up with respect to serum T-C, LDL-C, HDL-C, and LDL-C/HDL-C ratio (Table 2). There were no significant differences even when the combined VD, VD-Ca and MMN-VD-Ca group was compared with the PL group or when analyzing separately using changes in S-25OHD levels as covariate (data not shown). We observed a significant (p<0.05) positive effect on VLDL-C and TAG after supplementation. Percentages of subjects in different groups with serum lipids above and below the desirable range at baseline and after one year are presented in Table 3.

**Table 1.** Baseline characteristics of the randomly-assigned groups and changes from baseline characteristics after one year

Baseline physical and biochemical characteristics	Placebo (n=35)		VD (n=40)		VD-Ca (n=41)		MMN-VD-Ca (n=37)		p value
	Mean	SD	Mean	SD	Mean	SD	Mean	SD	
Age (years)	22.9	3.9	22.1	3.9	23.0	3.6	22.4	3.3	0.564
BMI (kg/m^2^)	21.7	2.4	22.0	2.8	21.3	3.1	21.5	2.6	0.606
HDL-C (mmol/L)	0.78	0.25	0.74	0.18	0.71	0.15	0.79	0.24	0.280
LDL-C (mmol/L)	2.24	0.82	2.30	0.70	2.29	0.54	2.27	0.58	0.543
LDL-C/HDL-C ratio	3.31	1.10	3.25	1.22	3.38	1.13	3.12	1.31	0.450
VLDL-C (mmol/L)	0.57	0.28	0.60	0.30	0.55	0.32	0.50	0.16	0.816
TC (mmol/L)	4.00	0.96	4.00	0.86	3.86	0.73	3.79	0.63	0.598
TAG (mmol/L)	1.26	0.63	1.34	0.68	1.24	0.71	1.11	0.35	0.450

BMI=Body mass index; Ca=Calcium; HDL-C=High-density lipoprotein cholesterol; LDL-C=Low-density lipoprotein cholesterol; MMN-VD-Ca=Multiple micronutrients-Vitamin. D-Calcium; TAG=Triacylglycerol; TC=Total cholesterol; VD=Vitamin D; VD-Ca=Vitamin D-Calcium

**Table 2.** Change from baseline characteristics after one year

Baseline physical and biochemicalcharacteristics	Placebo (n=35)		VD (n=40)		VD-Ca (n=41)		MMN-VD-Ca (n=37)		p value
	Mean	SD	Mean	SD	Mean	SD	Mean	SD	
BMI (kg/m^2^)	-0.12	2.04	0.48	1.25	0.10	1.38	-0.06	1.49	0.324
HDL-C (mmol/L)	-0.03	0.24	-0.05	0.22	0.02	0.15	-0.03	0.21	0.726†
LDL-C (mmol/L)	-0.11	0.73	0.03	0.68	-0.05	0.43	-0.05	0.62	0.826
LDL-C/HDL-C ratio	0.05	1.10	0.25	0.94	-0.09	0.89	-0.04	1.08	0.364
VLDL-C (mmol/L)	-0.11	0.21	0.01	0.22	0.01	0.25	0.02	0.17	0.039
T-C (mmol/L)	-0.09	0.88	0.02	0.95	0.07	0.54	0.12	0.73	0.714‡
TAG (mmol/L)	-0.25	0.48	-0.02	0.51	0.03	0.57	0.04	0.38	0.048^¥^

BMI=Body mass index; HDL-C=High-density lipoprotein cholesterol; LDL-C=Low-density lipoprotein cholesterol; MMN-VD-Ca=Multiple micronutrients-Calcium; TAG=Triacylglycerol; TC=Total cholesterol; VD=Vitamin D; VD-Ca=Vitamin D-Calcium; Mean values were not significantly different from those in analysis of covariance using baseline value as covariate;

^†^p=0.726 and using baseline value and BMI as covariates;

^‡^p=0.714; Mean value was significantly different from those in analysis of covariance, using baseline value and BMI as covariates, p=0.048

**Figure 2.**
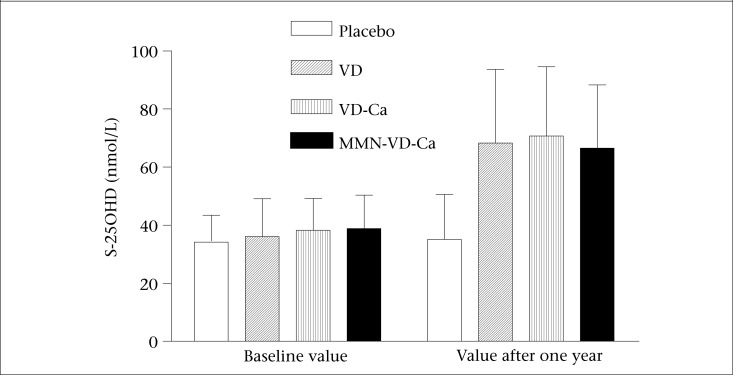
Serum 25-hydroxyvitamin D concentrations at baseline and after 12 months of supplementation

**Figure 3.**
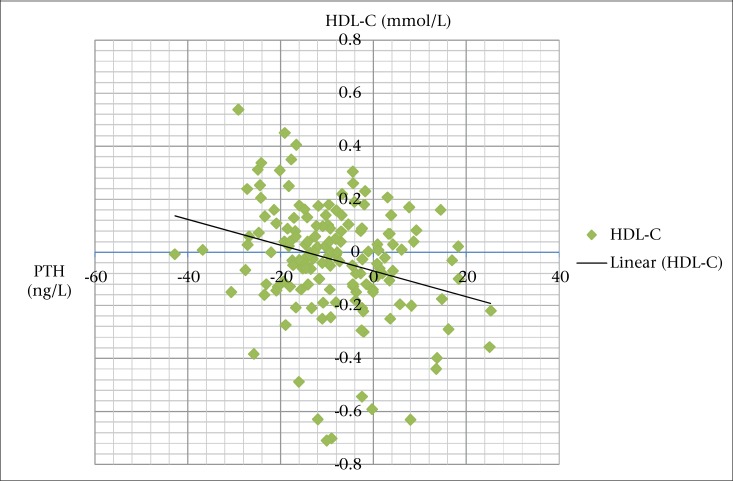
Negative correlation between changes in serum iPTH and HDL cholesterol levels

Significantly higher S-25OHD concentrations were observed in the supplemented groups than in the placebo group (Figure 2). Supplementation had an effect (p<0.001) on S-iPTH in the VD-Ca and MMN-VD-Ca groups compared to the placebo group, which is presented elsewhere (12). We observed a negative correlation between changes in serum iPTH and HDL-C. Our results indicated that subjects with the greatest decline in S-iPTH had the greatest increase in HDL-C (Figure 3).

## DISCUSSION

Vitamin D is integral to numerous physiologic functions in cells and tissues. Several studies reported the effect of vitamin D supplementation on cardiovascular risk factors in elderly subjects (14-21). The findings of all of these studies are inconsistent. A few studies have examined the effect of vitamin D supplementation on lipid profile among young women of childbearing age. The present study investigated the possible impact of vitamin D supplementation on lipid profile among pre-menopausal women with hypovitaminosis D. The study among Finnish post-menopausal women suggested the unfavourable effect of vitamin D supplementation on serum lipids. The study observed that LDL-C significantly increased by 4.1% and HDL-C decreased by 5.2% with daily supplement of 7.5 µg of vitamin D for 3 years (16). On the contrary, our results did not indicate any beneficial (nor detrimental) effects of supplementation on serum lipid-lipoprotein concentrations in apparently healthy young women with hypovitaminosis D. Our findings are consistent with several other studies on vitamin D intervention that continued for one year or less than one year and did not find any effect on blood lipids and lipoproteins (21-24). However, the unfavourable or detrimental effect of supplementation that continued further than one year is still controversial (25-27).

**Table 3.** Percentages of subjects in randomly-assigned groups with serum lipid variables below and above predeﬁned cutoffs (at baseline and after one year)

Serum lipid	At baseline				After one year			
	Placebo (n=35)	VD (n=40)	VD-Ca (n=41)	MMN-VD-Ca (n=37)	Placebo (n=35)	VD (n=40)	VD-Ca (n=41)	MMN-VD-Ca (n=37)
HDL-C <1.0 mmol/L	80.0	95.0	95.1	83.8	85.7	92.5	97.5	89.2
LDL-C >3.0 mmol/L	17.1	20.0	12.2	13.5	14.3	15.0	7.3	16.2
T-C >5.0 mmol/L	8.6	15	4.9	2.7	5.7	12.5	7.3	13.5
TAG >2.0 mmol/L	14.2	17.5	12.2	-	2.6	12.5	12.2	-

Ca=Calcium; HDL-C=High-density lipoprotein cholesterol; LDL-C=Low-density lipoprotein cholesterol; MMN-Ca=Multiple micronutrients-Vitamin D-Calcium; TAG=Triacylglycerol; TC=Total cholesterol; VD=Vitamin D; VD-Ca=Vitamin D-Calcium

Serum lipid is one of the major risk factors of CVD. The present study was designed to illustrate the possible effects of vitamin D supplementation with or without calcium and vitamin D with multiple micronutrients in subjects with hypovitaminosis D resulting from an exclusive indoor-working lifestyle and limited or no outdoor activities. No significant effects of supplementation for one year on any of the lipids were observed in any of the supplemented groups.

At baseline, we observed that serum HDL-C concentrations in the subjects were uniformly low (<1.0 mmol/L) in more than 80% of the subjects in different groups, and no significant changes were observed after one year of intervention, suggesting that vitamin D supplementation in recommended doses and with calcium or multiple micronutrients may have no influence on serum lipid and lipoprotein manipulation.

In fact, the present study was designed to examine the effect of vitamin D supplementation on skeletal health. Thus, the power was calculated with regard to the change in bone mineral content (12). Second, the doses given may have been sufficient for maintaining skeletal health but may have been insufficient for optimal effects on other health outcomes, like lipid profile. Recent reports suggested 2,000 IU to 10,000 IU of vitamin D per day as the safe doses and to have significant public health implications. The minimal target level of serum 25OHD should be above 75 nmol/L but 90-120 nmol/L could be favourable for optimal effects on health outcomes (28-30). We, therefore, believe that the result could have been different had we calculated the power for changes in serum lipids or used higher doses of vitamin D. A significant change was observed in serum 25OHD concentration after one year of intervention (Figure 2), although the mean values were much lower than the suggested minimal target level (≥75 nmol/L) for multiple health outcomes.

### Limitations

The findings could not be generalized for Bangladeshi women, even for all the female garment factory workers of Bangladesh, although the social background, dietary habit, and lifestyle of the garment factory workers may not differ significantly. However, results could be more representative by inclusion of subjects from different garment factories in Dhaka city and women from different socioeconomic status, or a post-menopausal group as the post-menopausal influences in serum lipid profiles might be associated with increased risk of coronary heart disease. Results could have been different if different doses of vitamin D supplement were used. In addition, due to some technical limitations, we could not measure dietary vitamin D intake and blood pressure in the subjects, which could add weight to the study.

### Conclusions

The recommended dietary intake of 10 µg of vitamin D/day with calcium or multiple micronutrients for one year may have no influence on serum lipid profile. The long-term supplementation of vitamin D and its adverse effect remain unclear and debatable. Therefore, longer-term clinical trial is warranted in evaluating the effect of intervention. Second, the present study indicated that recommended dose of dietary vitamin D intake (10 µg/day) might be not sufficient for young female garment factory workers with hypovitaminosis D to maintain adequate vitamin D status (serum 25-hydroxyvitamin D level >75 nmol/L).

## ACKNOWLEDGEMENTS

This study was supported by a research grant of the Development Fund of the Academy of Finland. We thank Beximco Pharmaceutical Ltd., Dhaka, Bangladesh, for providing MMN and Gonoshasthaya Pharmaceuticals Ltd., Dhaka, Bangladesh, for calcium preparations. The authors are grateful to Chief Medical Officer Dr. F.A. Al-Arif, officials of the Board of Directors of the Standard Garments Factory for their cooperation. We thank the 153 volunteer subjects who completed the study and made this research possible.

**Conflict of interest:** None of the contributing authors had any financial or personal conflicts of interest.
